# Young bodies, aging ovaries: A systematic review and meta‐synthesis of women's perspectives on spontaneous premature ovarian insufficiency

**DOI:** 10.1002/ijgo.71097

**Published:** 2026-06-12

**Authors:** Maria Patricia Roman, Shaurya Aggarwal, Stergios K. Doumouchtsis

**Affiliations:** ^1^ 2nd Department of Obstetrics and Gynaecology “Iuliu Hatieganu” University of Medicine and Pharmacy Cluj‐Napoca Romania; ^2^ 2nd Department of Obstetrics and Gynaecology Cluj County Clinical Hospital Cluj‐Napoca Romania; ^3^ Lister Hospital East and North Hertfordshire NHS Trust Stevenage UK; ^4^ Department of Obstetrics and Gynaecology Epsom and St Helier University Hospitals NHS Trust Epsom UK; ^5^ Institute of Medical and Biomedical Education St George's University of London London UK; ^6^ Laboratory of Experimental Surgery and Surgical Research “N.S. Christeas” National and Kapodistrian University of Athens, Medical School Athens Greece; ^7^ School of Medicine American University of the Caribbean Cupecoy Sint Maarten USA

**Keywords:** early menopause, meta‐synthesis, premature ovarian insufficiency, qualitative research, women's perspectives

## Abstract

**Objectives:**

Spontaneous premature ovarian insufficiency (POI) is a condition characterized by the cessation of ovarian function before age 40, unrelated to medical interventions. It affects approximately 3.5% of women worldwide and can result in significant emotional, physical, and social consequences. Despite clinical advancements in understanding POI, women's lived experiences remain underexplored, particularly the impact of early reproductive aging that conflicts with their chronological youth. This study aims to synthesize qualitative evidence on women's experiences of spontaneous POI, focusing on emotional, physical, social, and diagnostic challenges, as well as coping strategies.

**Method:**

A comprehensive search was conducted in MEDLINE, Embase, Scopus, Google Scholar, CINAHL, and PsycINFO from inception to January 2026, with studies screened independently by two reviewers using predefined criteria. Included studies employed qualitative methodologies, were peer‐reviewed, and were published in English. Studies on iatrogenic POI or using mixed/quantitative methods were excluded. Participant characteristics were extracted using an adapted Cochrane data collection form. Two researchers independently performed line‐by‐line coding using Dedoose software, resolving discrepancies via team discussion. Themes were developed through inductive analysis.

**Results:**

Thirteen studies involving 389 women revealed five key themes: (1) Emotional and psychological impact; (2) Physical and embodied experiences; (3) Social and relational challenges; (4) Diagnostic and treatment challenges; and (5) Coping and adaptation.

**Conclusion:**

Spontaneous POI profoundly affects women biopsychosocially, beyond reproductive loss. Clinical practice must shift toward woman‐centered care, integrating emotional support, timely diagnosis, and culturally sensitive interventions.

## 1 INTRODUCTION

### 1.1 Background

Premature ovarian insufficiency (POI), previously referred to as premature ovarian failure,^1^, ^2^ refers to the cessation of ovarian function before the age of 40, secondary to follicular dysfunction or depletion.^3^, ^4^, ^5^ Affecting approximately 3.5% of women globally,^6^ POI stems from diverse causes, including genetic conditions, autoimmune disorders, or idiopathic mechanisms.^7^, ^8^, ^9^ While medical interventions including chemotherapy and oophorectomy account for a subset of cases, the present meta‐synthesis specifically examines spontaneous POI, defined by its non‐iatrogenic etiology.

The premature loss of reproductive function generates a biological paradox, in which women experience reproductive aging typically associated with midlife, while maintaining their social and psychological youth. This temporal dissonance, the sensation of being simultaneously young and old, defies traditional age norms, potentially unsettling one's sense of identity, fertility aspirations, and social cohesion.^10^, ^11^ Studies have shown that this deviation from anticipated reproductive timelines can trigger significant psychological distress, frequently surpassing the intensity of emotions associated with the course of natural menopause.^12^ The grief of unfulfilled motherhood, altered body image and profound feelings of inadequacy are frequently intensified by a lack of robust socio‐familial support^13^ and limited acknowledgment by clinicians.^10^

Previous quantitative studies investigated the clinical characteristics and treatment options for POI^14^, ^15^, ^16^; however, qualitative research provides critical insights into women's lived experiences of diagnosis, their coping strategies, and their identity reconstruction in light of reproductive loss. While individual qualitative studies have advanced our understanding of these issues, there is a lack of comprehensive syntheses that encapsulate the breadth and depth of women's lived experiences with spontaneous POI. Unlike iatrogenic POI, which typically follows a known medical intervention such as chemotherapy or oophorectomy, spontaneous POI occurs without an external precipitant and is frequently characterized by prolonged diagnostic uncertainty and unexpected reproductive loss. This difference appears to shape women's emotional responses, with spontaneous POI being more strongly associated with shock, confusion, and mistrust of bodily signals, whereas iatrogenic POI might be experienced within a narrative of survival and medical necessity. Further, the psychosocial consequences of discordant reproductive aging, where biological and chronological ages diverge, remain insufficiently explored, especially as they interplay with sociocultural norms of femininity, fertility plans, and life‐course trajectories.^17^, ^18^

### 1.2 Objectives

This study aimed to synthesize qualitative evidence on women's lived experiences of spontaneous POI, developing and exploring key themes from the perspective of women navigating the condition. By integrating and critically analyzing existing qualitative research, we aimed to deepen theoretical understanding while uncovering actionable insights to inform women‐centered clinical practice. Ultimately, this work seeks to bridge gaps between patient experiences and healthcare delivery, fostering improved support and tailored interventions for those affected by POI.

## 2 METHODS

### 2.1 Study registration

This study was registered with the International Prospective Register of Systematic Reviews (PROSPERO), registration number CRD420250655495, and adheres to the Enhancing Transparency in Reporting the Synthesis of Qualitative Research (ENTREQ) statement (Material S1).^19^

### 2.2 Search strategy and selection of studies

The search for this meta‐synthesis was pre‐planned, involving comprehensive strategies to identify all available qualitative primary studies relevant to women's lived experiences of spontaneous POI. This approach involved systematically searching MEDLINE, Embase, Scopus, Google Scholar, CINAHL, and PsycINFO databases, from their inception to January 2026, using a structured combination of keywords (Appendix S1). The pre‐planned strategy aimed to ensure a thorough and reproducible retrieval of studies, minimizing bias and capturing the full spectrum of existing evidence. This method aligns with the systematic nature of the review, prioritizing breadth and transparency, given the study's goal of synthesizing a defined body of qualitative findings to inform clinical practice.

The research team defined specific inclusion criteria to ensure study relevance and rigor. Studies were included if they employed a purely qualitative methodology and were published in English, in peer‐reviewed journals. Studies were excluded if they used mixed or quantitative methodologies, if they were dissertations or theses that had not undergone a peer‐review process, or if they investigated iatrogenic causes of POI. These exclusions ensured a focus on rigorously vetted qualitative research and a higher standard of methodological scrutiny.

The screening of studies followed a systematic, two‐stage assessment process to ensure adequate selection. Initially, the titles and abstracts of all identified records were independently reviewed by two researchers (MPR and SA) against the predefined inclusion criteria mentioned above. Studies meeting those criteria advanced to full text review, where the same two reviewers assessed eligibility in greater detail, resolving discrepancies through discussion within the research team. This dual‐reviewer approach minimized bias and enhanced reliability. Snowballing was used to identify potentially eligible additional articles, by examining the reference lists of included studies and relevant reviews. The study selection process was tracked using a PRISMA flow chart (Appendix S2), documenting the number of studies screened, excluded, and ultimately included.

### 2.3 Data extraction

The following study characteristics were extracted from the primary studies: study aim, geographic location, sample size, source of the study sample, data collection method, data analysis method, and whether data saturation was achieved. Data were extracted using a modified version of the Cochrane data collection form for intervention reviews (randomized controlled trials [RCTs] and non‐RCTs),^20^ as detailed in Appendix S3.

### 2.4 Data synthesis

Thematic analysis was used to synthesize qualitative findings from the included studies. This approach was chosen for its flexibility in handling diverse qualitative data and its capacity to balance fidelity to original findings with the generation of new, cross‐study understandings. The analyses involved systematically, independently coding of data, line‐by‐line, by two researchers, to identify recurring patterns and concepts using Dedoose software (version 10.0.25).^21^ The codes were then grouped into categories, based on conceptual similarities, reflecting the content of the primary studies. These categories were further refined into analytical themes, offering deeper interpretive insights into women's lived experiences of spontaneous POI. Patient quotations were extracted to illustrate and support the emerging themes.

Sensitivity mapping of codes to their originating study methodology was conducted to address methodological heterogeneity across included studies (hermeneutic phenomenology, thematic analysis, content analysis, and narrative approaches).

### 2.5 Risk of bias assessment

Assessing the quality and risk of bias in studies strengthens the methodological rigor.^22^ Common biases in qualitative research include interviewer, procedural, measurement, sampling, design, response, and reporting biases, among others. Given the lack of international consensus on a single best appraisal tool,^23^ we selected the Critical Appraisal Skills Programme (CASP)^24^ for the assessment of the included studies. This tool was developed to help researchers and healthcare professionals systematically evaluate the relevance and methodological rigor of qualitative research. It features 10 key questions that assess study validity, data collection, analysis, and applicability, ensuring a structured approach to critical appraisal. Widely endorsed by organizations such as Cochrane,^25^ it supports novice and experienced researchers in evidence‐based practice by identifying strengths, limitations, and biases in qualitative studies, with a focus on improving the quality of evidence synthesis in health and social care. Two researchers independently conducted the evaluation using the CASP tool for qualitative studies, resolving discrepancies through discussion.

The research team consisted of researchers with a background in women's health and qualitative research with a heightened sensitivity to the challenges of POI described by participants. However, due to the potential influence of our backgrounds and perspectives on the research findings and interpretations, the risk of bias should be acknowledged. To mitigate it, we engaged in ongoing reflexive practices throughout the study. An independent coding process allowed us to cross‐check the interpretations, while regular discussions enabled us to resolve discrepancies related to coding, theme development and the quality appraisal of included studies. These strategies were implemented to ensure that our perspectives did not unduly shape the findings.

## 3 RESULTS

### 3.1 Study characteristics

This meta‐synthesis included 13 qualitative studies involving 389 women with spontaneous POI, from diverse geographic locations. The studies employed various qualitative methodologies, such as hermeneutic phenomenology, thematic analysis, and content analysis, to explore women's lived experiences of POI. Table 1 includes the main characteristics of the included studies.

**TABLE 1:** Characteristics of primary studies.

First author, year of publication	Sample size (*N*)	Country where study was conducted	Recruitment source	Data collection method	Data analysis method	Data saturation
Boughton et al., 2002	35	Australia (multicentric study)	Women responding to an advertisement in newspaper articles/a radio announcement, menopause support center	Face‐to‐face interviews, collaborative interviews	Hermeneutic phenomenology	Not specified
Boughton et al., 2008	35	Australia (multicentric study)	Women responding to an advertisement in newspaper article/a radio announcement, menopause support center	Face‐to‐face interviews, collaborative interviews	Hermeneutic phenomenology, thematic analysis	Not specified
Golezar et al., 2020	16	Iran	Gynecology clinic	Semi‐structured in‐depth interviews	Conventional content analysis	Achieved
Grootenhuis et al., 2021	15	The Netherlands	Educational institutions	Semi‐structured face‐to‐face interviews	Thematic analysis with an interpretive approach	Achieved
Halliday et al., 2009	98	USA, UK, Canada, Australia, South Africa, Hungary, Ireland, and Sri Lanka	Online women's health discussion board	Analysis of online discussion board messages	Thematic analysis	Not specified
Johnston‐Ataata et al., 2020	25	Australia	Specialist early menopause/POI clinics, cancer support/research organizations, universities, health promotion groups, professional clinical societies	Narrative semi‐structured interview	Thematic narrative analysis	Not specified
Johnston‐Ataata et al., 2020	30	Australia	Specialist early menopause/POI clinics, cancer support/research organizations, universities, health promotion groups, professional clinical societies	Narrative interviews	Thematic analysis	Not specified
Moukhah et al., 2021	16	Iran	Infertility centers	Semi‐structured in‐depth interviews	Content analysis	Achieved
Moukhah et al., 2023	15	Iran	Fertility center, research Institute	In‐depth and semi‐structured interviews	Content analysis	Achieved
Orshan et al., 2001	6	USA	Metabolic testing unit	Interviews	Colaizzi's procedure	Not specified
Singer et al., 1999	25	UK	Clinical networks	Semi‐structured focused interviews	Thematic discourse analysis	Not specified
Sun et al., 2022	43	China	Outpatient clinic	In‐depth, semi‐structured interviews	Thematic analysis	Achieved
Vincent et al., 2024	30	Australia	Outpatient clinic	In depth narrative interviews	Dual: thematic and thematic narrative analysis	Achieved
	Total 389					

Abbreviation: POI, premature ovarian insufficiency.

To understand the context of women's perspectives on spontaneous POI, we examined the characteristics of participants across the 13 included studies. These characteristics, including reproductive history, relationship status, sexual orientation, education level, and occupation, provide insight into the diverse backgrounds of the women whose experiences were explored. Table 2 summarizes these characteristics, highlighting the variability among participants.

**TABLE 2:** Participant characteristics of included studies.

Study	Reproductive history	Relationship status	Sexual orientation	Education level	Professional status
Boughton et al., 2002	Previous pregnancies: 77.1% None: 22.9%	Married: 65.7%	Heterosexual: 100%	Not specified	Not specified
Boughton et al., 2008	Not specified	Not specified	Heterosexual: 100%	Not specified	Not specified
Golezar et al., 2020	Previous pregnancies:37.5% None: 62.5%	Single: 25% Married: 62.5% Divorced: 12.5%	Not specified	Primary: 6.2% Undergraduate: 25% Associate's degree: 6.2% Bachelor's degree: 37.5% Master's degree: 25%	Student: 12.5% Employed: 43.7% Unemployed: 43.7%
Grootenhuis et al., 2021	Not specified	Single: 40% Partnership: 60%	Heterosexual: 93.3% Other: 6.7%	Medium professional school: 33.3% Higher professional school: 33.3% University: 33.3%	Employed: 66.7% Unemployed: 33.3%
Halliday et al., 2009	Previous pregnancies: 43% None: 31% Unknown: 26%	Married: 59% Partnership: 2% Divorced: 1% Unknown: 38%	Not specified	Not specified	Not specified
Johnston‐Ataata et al., 2020	Previous pregnancies: 48% Pregnant (self or partner): 7% None: 44%	Single: 32% Partnership: 68%	Heterosexual: 76% Other: 20% Not stated: 4%	Secondary: 8% Post‐secondary: 92%	Employed: 84% Unemployed: 16%
Johnston‐Ataata et al., 2020	Previous pregnancies: 46.7% Pregnant (self or partner): 6.7% None: 46.7%	Single: 30% Partnership: 70%	Heterosexual: 76.7% Other: 20% Not stated: 3.3%	Not specified	Not specified
Moukhah et al., 2021	Previous pregnancies: 56.2% None: 43.7%	Married: 100%	Not specified	Primary: 31.2% Undergraduate: 31.2% Bachelor's degree: 12.5% Master's degree: 12.5% PhD degree: 12.5%	Employed: 31.2% Unemployed: 68.7%
Moukhah et al., 2023	Previous pregnancies: 53.3% None: 46.6%	Married: 86.7% Divorced: 13.3%	Not specified	Primary: 26.6% Undergraduate: 33.3% Bachelor's degree: 13.3% Master's degree: 13.3% Ph.D. degree: 13.3%	Employed: 33.3% Unemployed: 66.6%
Orshan et al., 2001	Previous pregnancies: 50% (all through egg donation) None: 50%	Single: 33.3% Married: 50% Divorced:16.6%	Heterosexual: 100%	Not specified	Not specified
Singer et al., 1999	Children (including adoption): 61.5% No children: 38.5%	Single: 46.1% Married: 38.4% Divorced: 15.3%	Not specified	Not specified	Not specified
Sun et al., 2022	Children: 65.12% No children: 34.8%	Single: 13.9% Married: 72% Divorced:13.9%	Not specified	Less than high school: 4.6% High school: 25.5% College: 60.4% Graduate degree: 9.3%	Student: 2.3% Employed: 93% Unemployed: 4.6%
Vincent et al., 2024	Previous pregnancies: 46.7% Pregnant (self or partner): 6.7%	Single: 30% Partnership: 70%	Heterosexual: 76.6% Other: 20% Unknown: 3.3%	High school: 13.3% Diploma: 10% University degree: 76.6%	Employed: 83.3% Unemployed: 16.6%

Abbreviation: POI, premature ovarian insufficiency.

Participants' ages varied widely, from adolescence to mid‐adulthood, with a mean of 32 years, underscoring the diverse life stages at which women might experience POI. Reproductive history also showed significant variation, with some women having previous pregnancies or children (including through adoption or egg donation), while others had no children, which might influence their perspectives on fertility and family‐building in the context of POI. Relationship status ranged from single to married/divorced or in partnerships, with most studies (11/13) noting a majority of participants in relationships, potentially enhancing their social support systems. Sexual orientation was predominantly heterosexual where reported, although a few studies included participants identifying as other orientations (homosexual or bisexual), adding to the diversity of experiences. Education levels varied across studies, ranging from high rates of post‐graduate education to lower educational attainment among participants. Most participants were employed, underscoring that many were in an active life stage when confronting POI. This demographic diversity highlights the importance of considering how social and educational backgrounds might influence women's experiences of POI.

Six of the 13 selected studies included participants with medically induced POI; however, in each case, the findings pertaining to women with spontaneous POI were clearly distinguishable from those with induced POI. To maintain methodological consistency and uphold the homogeneity principle of this synthesis, our analysis exclusively focused on data derived from women with spontaneous POI.

Each study explored various dimensions of women's experiences with POI. To provide an overview of the scope of these studies, Table 3 presents the included studies along with their specific aims. The aims reflect a diverse range of perspectives, from understanding the emotional and social consequences of POI to examining its effects on identity, relationships, and workplace experiences. These studies collectively informed the development of the analytical themes.

**TABLE 3:** Included studies and their aims.

First author, year of publication	Aims
Boughton et al., 2002	“To explore the experience of menopause among women under the age of 40, while considering how women's bodies undergo changes with age and the societal expectations tied to age‐appropriate appearances and representations.”^18^
Boughton et al., 2008	“To find an explanation for the physical and emotional symptoms the women were experiencing.”^26^
Golezar et al., 2020	“To highlight the factors affecting the quality of life of women with POI.”^12^
Grootenhuis et al., 2021	“To explore young women's opinions about predicting premature ovarian failure using an AMH blood test, focusing on perspectives across educational levels.”^27^
Halliday et al., 2009	“To establish which aspects of being a younger menopausal woman proved problematic and to determine if the cause of premature menopause altered the experience.”^28^
Johnston‐Ataata et al., 2020	“To explore how early menopause or POI affects personal relationships and contributes to the formation of a new subjectivity, using concepts of biographical disruption and social recognition.”^11^
Johnston‐Ataata et al., 2020	“To generate a comprehensive understanding of women's experiences of early menopause/POI encompassing its medical, social, and emotional dimensions.”^29^
Moukhah et al., 2021	“To investigate perception and experience of women with POI of sexual and reproductive health.”^30^
Moukhah et al., 2023	“To investigate the perception and experience of women with POI about female identity.”^13^
Orshan et al., 2001	“To describe the lived experience of women with idiopathic premature ovarian failure.”^31^
Singer et al., 1999	“To explore women's lived experiences of premature menopause and how these experiences relate to medical and gendered discourses of aging, reproduction, and identity.”^10^
Sun et al., 2022	“To explore the chronic and cumulative adverse life events experienced by women with POI.”^32^
Vincent et al., 2024	“To explore the experiences of women with early menopause or POI in relation to work.”^17^

### 3.2 Thematic synthesis

Following independent line‐by‐line coding by two researchers and subsequent centralization, 35 codes were identified (Figure 1). The process of deriving codes from the excerpts was inductive, given that no pre‐existing theoretical framework was used to inform the coding process. The heatmap (Figure 1) visually represents the frequency and distribution of the codes across the included studies, highlighting the prominence of reproductive loss, psychosocial challenges, and adaptation efforts in women's lived experiences of POI. The intensity of color corresponds to the prevalence of each code. We note that code frequency does not equate to conceptual importance in qualitative synthesis. The heatmap serves a descriptive purpose, illustrating code distribution across studies, and does not imply that more frequent codes are inherently more significant. Thematic development was guided by contextual meaning, not by frequency alone.

**FIGURE 1:**
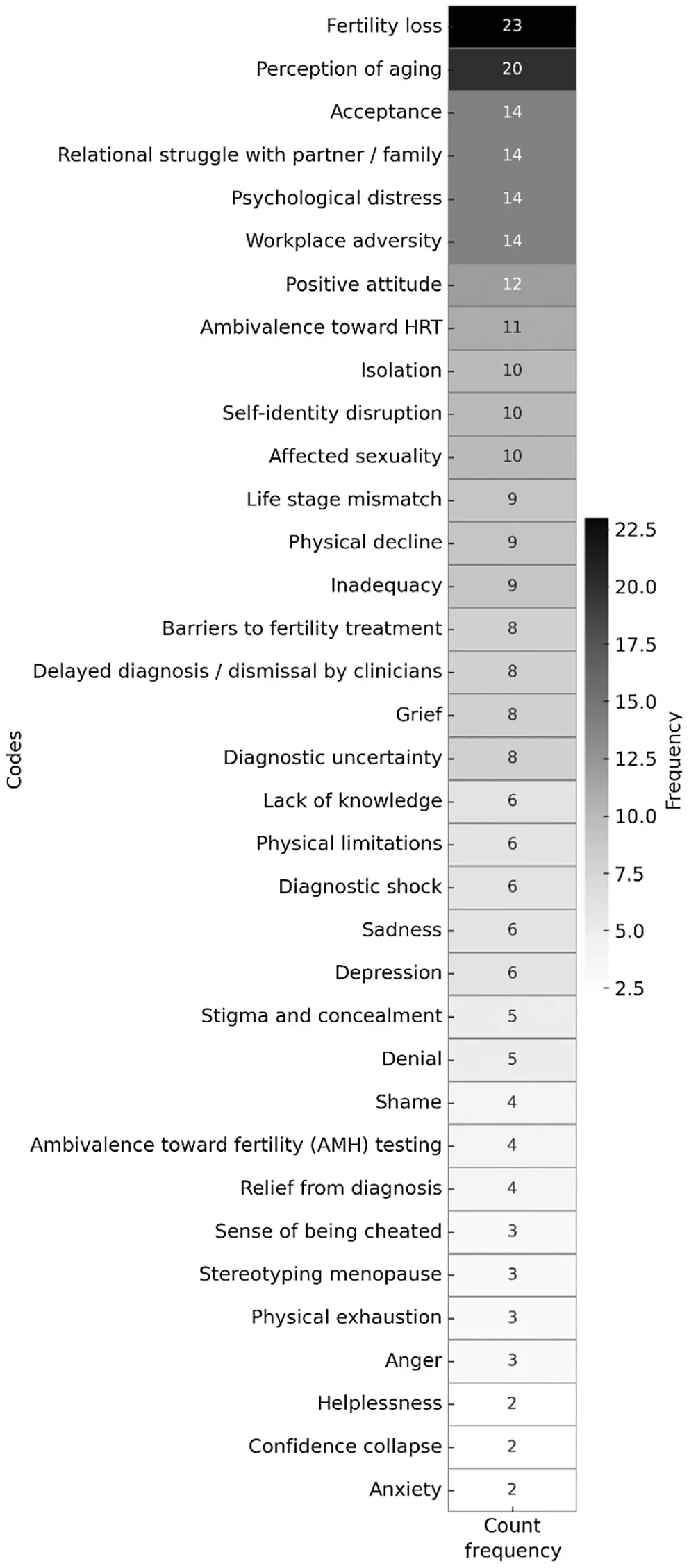
Heatmap of codes.

These codes were grouped into five analytical themes that capture the multifaceted impact of POI on women's lives: (1) Emotional and psychological impact, (2) Physical and embodied experiences, (3) Social and relational challenges, (4) Diagnostic and treatment challenges, and (5) Coping and adaptation. Appendix S4 presents the analytical themes, the contributing codes, and relevant patients' quotations from the primary studies. Participant quotations provide direct insight into lived experiences, reflecting the diversity and depth of perspectives on living with POI.

The following sections provide a deeper analysis of each theme, starting with the “Emotional and psychological impact of POI.” This theme captures the psychological toll of POI, highlighting negative emotions, identity challenges, and societal perceptions that exacerbate distress. Contributing codes such as “Depression” and “Grief” directly address mental health struggles, while “Feeling diminished by POI,” “Sense of being cheated” and “Self‐identity disruption” reflect the impact on self‐perception and identity. “Stigma and concealment” reflect societal attitudes that contribute to emotional distress, leading to “Shame” buildup.

The second theme, “Physical and embodied experiences,” focuses on changes in the body and their impact on daily life, sexuality, and reproductive capacity. Contributing codes “Perception of aging” and “Physical decline” reflect changes like premature aging and bodily deterioration, while “Physical limitations” and “Physical exhaustion” highlight functional impacts. “Affected sexuality” captures the intimate consequences of POI, and “Fertility loss” underscores the embodied experience of losing reproductive potential, making these key aspects of the lived experience.

The third theme, “Social and relational challenges,” encompasses the social and relational difficulties that arise from POI, including impacts on relationships, work, and societal expectations. The codes “Relational struggle with partner/family” and “Workplace adversity” address challenges in personal and professional relationships, while “Life stage mismatch” captures the disconnect between societal expectations and the participant's life stage. “Stereotyping menopause” reflects societal attitudes that exacerbate social challenges, such as assumptions about aging and femininity, often leading to “Isolation.”

The fourth theme, “Diagnostic and treatment challenges” centers on the difficulties surrounding the diagnosis of POI and the ambivalence toward treatment options, highlighting barriers to care and understanding. Codes such as “Diagnostic uncertainty,” “Delayed diagnosis/dismissal by clinicians,” and “Diagnostic shock” reflect issues in the diagnostic process, while “Lack of knowledge” underscores gaps in understanding. The codes “Ambivalence toward Anti‐Müllerian Hormone (AMH) Testing” and “Ambivalence toward Hormone Replacement Therapy (HRT)” reflect women's mixed feelings about screening for POI and its treatment options, respectively, while “Barriers to Fertility Treatment” underscores the challenges in overcoming infertility.

The final theme, “Coping and Adaptation,” captures the strategies women employ to manage and adjust to life with POI, highlighting their capacity for resilience and positive reframing. The codes “Positive attitude” and “Acceptance” reflect how women navigate their experiences by finding new meaning and ways to move forward. Additionally, the code “Relief from diagnosis” underscores the sense of reassurance and clarity that a definitive diagnosis can provide, offering emotional validation and supporting the coping process.

All five analytical themes are supported by four distinct qualitative methodologies (hermeneutic phenomenology, thematic analysis, content analysis, and narrative analysis), indicating that no theme relies exclusively on a single methodological approach. This cross‐method convergence strengthens the credibility of the thematic synthesis. Detailed mapping of codes to themes and contributing studies is presented in Appendix S4, while the methodological characteristics of each included study are reported in Table 1.

### 3.3 Risk of bias assessment

To ensure methodological rigor, all included studies were evaluated using the CASP checklist for qualitative research. Most studies (11/13) met ≥8 of the 10 CASP criteria, demonstrating clear research aims, appropriate qualitative methodology, and rigorous data collection and analysis. While 12 studies explicitly addressed ethical approval, one study (Orshan et al.^31^) lacked documentation of ethical considerations. Four studies did not describe how researcher bias or relationships with participants were addressed, while in five additional studies, it was unclear whether this criterion had been met (labeled as “can't tell” in the CASP assessment). The remaining four studies fully met the CASP criterion regarding researcher–participant relationships. The CASP assessments are presented in Appendix S5.

Two assessors (MPR and SA) achieved a percentage agreement of 95.4% and a Cohen's Kappa of 0.68, indicating substantial inter‐rater agreement in their CASP quality appraisals. Discrepancies were reviewed with a third researcher (SKD), and a consensus was reached.

## 4 DISCUSSION

### 4.1 Main findings

Spontaneous POI poses a significant burden for young, active women. Although randomized controlled trials^33^, ^34^ and comparative cohort studies^35^, ^36^, ^37^, ^38^ have advanced our understanding of POI, including risk profiling^39^ and underlying genetic, autoimmune and other pathogenetic mechanisms, the lived experiences of women undergoing this transition remain underexplored.

To address this gap, this meta‐synthesis integrates findings from 13 qualitative studies involving 389 women with spontaneous POI, revealing the condition's profound and multifaceted impact. Through thematic synthesis, five key analytical themes emerged: “Emotional and psychological impact,” “Physical and embodied experiences,” “Social and relational challenges,” “Diagnostic and treatment barriers,” and “Coping and adaptation.” Together, these themes underscore the complex interplay of biological, psychological, and social factors shaping women's experiences of POI.

Emotionally, women frequently experience grief, depression, and self‐identity disruption, often exacerbated by societal stigma and stereotypes associating menopause with aging, which conflicts with their youthful chronological age.^10^ Physically, women report premature aging, fatigue, and fertility loss, which not only affect their daily functioning but also challenge their sense of femininity and reproductive potential. Socially, POI strains relationships and workplace dynamics,^17^, ^18^, ^26^ with many women feeling isolated due to a perceived life stage mismatch, as their reproductive aging does not align with societal expectations for their age.

Our meta‐synthesis identified persistent themes of diagnostic delay, clinician dismissal, and fragmented care across studies published between 1999 and 2024. It is important to contextualize these findings within the evolving clinical guidance landscape. The 2015 ESHRE guideline on POI management, which represented the standard of care for part of the study period, recommended two elevated follicle‐stimulating hormone (FSH) measurements for diagnosis and did not provide explicit guidance on the role of AMH testing, the importance of sensitive diagnosis disclosure, or standardized psychological support pathways.^40^ The absence of such guidance might have contributed to the diagnostic uncertainty and inconsistent care documented in some of the primary studies.

The newly published 2024 evidence‐based, collaborative guideline (Panay et al.^41^) addresses many of these gaps. Relative to the 2015 version, notable advances include: (i) revised diagnostic criteria requiring only one elevated FSH (>25 IU/L) to facilitate diagnosis; (ii) explicit guidance on compassionate diagnosis disclosure, shared decision‐making and referral to appropriate care; (iii) recognition that non‐use of HT is associated with increased cardiovascular mortality; (iv) recommendations for annual monitoring of cardiovascular risk factors; and (v) emphasis on multidisciplinary, woman‐centered care. We endorse these recommendations and recognize that their systematic implementation might reduce the diagnostic delays and fragmented care described by women in the included studies.

However, the persistence of these recurrent issues even in studies published after 2015 suggests that guideline dissemination alone is insufficient; implementation strategies and healthcare system redesign are equally essential. These systemic gaps align with findings by Jarcevic et al.^42^ who identified limited trainee exposure to POI and highlighted opportunities to strengthen education and clinical training, including the potential role of specialized clinics in improving awareness and experience with POI care. Moreover, educational interventions should be tailored to clinician type. For primary care and front‐line clinicians, training should emphasize diagnostic suspicion for POI and avoidance of dismissive language. For gynecologists, POI‐specific communication strategies should address prior invalidation, the age‐reproduction paradox, and proactive fertility discussions.

The 13 studies included in this meta‐synthesis were published between 1999 and 2024, with five of them conducted in the early 2000s or even before, prior to recent advances in ovarian reserve testing, genetic diagnostics, and standardized management guidelines. While our search extended to January 2026, the available qualitative literature captures diagnostic experiences from an era when AMH testing was less widely integrated into clinical practice, clinician awareness was lower, and standardized diagnostic criteria were less consistently applied. Consequently, themes such as “diagnostic delay,” “dismissal by clinicians,” and “prolonged uncertainty” might reflect outdated care pathways that are improving, although contemporary data are insufficient to confirm whether these delays have substantively diminished.

Further, the psychological impacts such as grief, identity disruption, and stigma remain highly relevant regardless of diagnostic timeliness,^43^, ^44^, ^45^ as the fundamental biopsychosocial burden of receiving a POI diagnosis at a young age persists. The coping and adaptation theme offers a counterbalance, suggesting that even within older studies, women demonstrated resilience. Future qualitative research is urgently needed to capture the experiences of women diagnosed in the current era of improved ovarian reserve biomarkers, improved diagnostic pathways, and evolving fertility preservation options to determine whether the distress of diagnostic delay has been meaningfully reduced or merely shifted to other phases of the POI experience.

Once diagnosed, women face new uncertainties surrounding treatment. Treatment options, such as HRT and fertility testing, elicit ambivalence, with women weighing benefits against side effects and cultural or religious barriers. Despite these challenges, some women demonstrate resilience through coping strategies, finding relief in a clear diagnosis and adopting a positive attitude or acceptance of their condition. These findings underscore the need for a holistic approach to POI care that addresses not only medical needs but also the emotional, social, and cultural dimensions of the condition.

### 4.2 Strengths and limitations

This meta‐synthesis demonstrates robust methodological rigor through its adherence to ENTREQ guidelines, dual‐reviewer coding, and CASP quality assessment. The substantial inter‐rater agreement (Cohen's kappa = 0.68; percentage agreement = 95.4%) between assessors MPR and SA in their CASP quality appraisals underscores the reliability of this evaluation, with discrepancies effectively resolved through consensus within the research team, enhancing the trustworthiness of our qualitative synthesis.

The deliberate focus on spontaneous POI (excluding medically induced cases) enhanced conceptual clarity by avoiding heterogeneity from differing etiologies (e.g., cancer treatment), which would have introduced confounding variables in psychological impacts and lived experiences. The inclusion of culturally, geographically, and sexually diverse participants provided rich intersectional insights, while researcher reflexivity strengthened interpretive validity. These design choices yielded a broad understanding of spontaneous POI's biopsychosocial dimensions.

While this synthesis provides valuable insights, several limitations warrant consideration. The predominance of studies from developed, high‐income countries (e.g., Australia, USA, and UK) might limit the transferability of findings to other cultural and healthcare contexts, where access to health care and cultural attitudes toward menopause might differ. For example, in patrilineal societies (e.g., parts of South Asia, Sub‐Saharan Africa, or the Middle East), POI might carry heightened risks of marital dissolution and stigmatization, whereas collectivist cultures might offer alternative coping resources through extended family networks. Access to fertility treatment also varies widely: where assisted reproduction is unavailable, grief might be compounded by absolute reproductive futility, unlike in countries like Australia, the USA, and the UK, where hope is often maintained. Cultural norms around disclosure further shape experiences, as privacy might exacerbate isolation, while community acknowledgment can facilitate support. Thus, these findings should be considered context bound, underscoring the need for primary qualitative research exploring POI experiences outside developed, high‐income countries like Australia, the USA, and the UK.

Second, the included studies predominantly recruited participants from menopause clinics, infertility centers, research institutions and online support communities. This might have introduced selection bias, as women who seek out such services likely experience greater emotional distress or exhibit stronger health‐seeking behaviors than those who do not. Consequently, our findings might better represent the experiences of help‐seeking women, while potentially missing the perspectives of those who face barriers to accessing care or are unaware of their diagnosis. Future research should purposively sample from community and primary care settings to capture a broader spectrum of POI experiences.

Finally, the exclusion of non‐English publications and gray literature constitutes a source of language bias, potentially overlooking culturally specific perspectives and potentially valuable unpublished data. In addition, variability in reporting participant characteristics across included studies constrained our ability to conduct more nuanced subgroup analyses, particularly regarding socioeconomic factors that might influence POI experiences. These gaps highlight the need for future research to include more diverse populations and standardized reporting of demographic data to enhance the comprehensiveness of findings.

### 4.3 Interpretation

These findings align with prior research on POI, which highlights its psychological toll, referring especially to feelings of anxiety and depression.^46^, ^47^ The emotional distress identified through codes such as “grief” and “self‐identity disruption” also resonates with Golezar et al.^12^ who noted that POI often triggers feelings of inadequacy and jealousy, particularly around fertility loss.

Notably, our results both converge and extend a recent thematic synthesis by Hammond and Marczak,^48^ who identified three analytical themes reflecting POI and related to diagnostic uncertainty, identity disruption, and support gaps. Our meta‐synthesis expands this framework by delineating five distinct themes that capture the biopsychosocial complexity of spontaneous POI. Specifically, we: broadened the emotional scope by integrating nuanced standalone dimensions such as “stigma and concealment,” a “sense of being cheated,” “helplessness” under “Emotional and psychological impact,” moving beyond diagnostic distress to capture the full affective burden pf POI. Additionally, we emphasized embodied experiences, such as premature aging and sexual dysfunction, which were underexplored in their synthesis.

By introducing resilience strategies (“Coping and adaptation”) as a central theme, we also balanced their predominantly deficit‐oriented perspective with evidence of women's adaptive strategies and post‐diagnostic growth. Adaptive strategies included cognitive reframing (viewing POI as a turning point rather than solely a loss), redefining womanhood beyond reproductive capacity, and seeking validation through peer support networks. Several women described post‐diagnostic growth manifested as increased self‐knowledge, advocacy for others with POI, or reorientation toward alternative family‐building or life goals. These strategies represent identity reconstruction rather than simple acceptance and suggest that psychological interventions could usefully target meaning‐making and self‐concept.

The physical and embodied experiences, such as fatigue and affected sexuality in women suffering from POI, mirror Pacello et al.^49^ who described the functional impact of ovarian insufficiency. However, our synthesis uniquely foregrounds the embodied sense of premature aging and its impact on self‐perception.

Contrary to findings from the case–control studies of Benetti‐Pinto et al.^46^, ^50^ which reported no significant differences in social relationships or quality of life domains among POI patients, this meta‐synthesis reveals substantial social ramifications. Our qualitative approach uncovers how societal stereotypes framing menopause as exclusively an “older woman's condition” uniquely exacerbate distress among women with POI, a dimension not captured in quantitative assessments of quality of life. This divergence highlights the value of qualitative methodologies in detecting nuanced psychosocial impacts that standardized instruments might overlook, particularly regarding identity disruption and social stigma. Additionally, this synthesis adds depth by identifying workplace adversity as a significant challenge, particularly for women in physically demanding roles, as noted by Vincent et al.^17^

Diagnostic challenges, such as “delayed diagnosis” and clinician dismissal, align with Moukhah et al.^30^ yet our synthesis uniquely emphasizes the emotional burden of “diagnostic shock,” an aspect underexplored in individual studies. The ambivalence surrounding HRT and fertility treatments reflects profound cultural and personal conflicts, challenging the uniform application of clinical guidelines that advocate for early intervention.^40^, ^41^ This tension underscores an urgent need to reconcile evidence‐based protocols with the diverse values and lived experiences of women navigating POI.

The coping and adaptation theme offers a counterpoint to the predominantly negative experiences, suggesting that a clear diagnosis can provide relief and facilitate acceptance, as reported by Boughton et al.^18^ This resilience aligns with broader qualitative literature on chronic illness, where understanding one's condition encourages empowerment.^51^ However, examination of coping strategies across different cultural contexts revealed notable variations. In Iranian studies,^12^, ^13^, ^30^ women frequently cited religious faith and fatalistic acceptance (“what God wills”) as primary coping resources, alongside greater reliance on family support networks. In contrast, Australian and UK studies emphasized individual psychological resilience, peer support groups, and active information‐seeking as dominant coping modes. These differences likely reflect broader cultural orientations toward illness, with collectivist societies mobilizing family‐based coping and individualist societies favoring self‐directed strategies. Importantly, as previously mentioned, cross‐cultural convergence was also observed: women across all settings reported relief at receiving a definitive diagnosis and valued having their symptoms validated by healthcare providers. These findings suggest that while the expression of coping might be culturally shaped, the fundamental need for diagnostic clarity and emotional validation might be universal. Clinicians should therefore assess and support coping resources that align with each woman's cultural framework rather than imposing a single model of adaptation.

The code frequency heatmap complements thematic synthesis by visually displaying the relative frequency of codes across studies. It underscores the salience of fertility loss, perceived premature aging, and psychological distress in women's accounts of spontaneous POI. Frequent references to relational disruption and workplace challenges further highlight the condition's broader social repercussions. Notably, fewer references to adaptive responses such as positive attitude or relief from diagnosis suggest that coping and resilience remain underexplored areas within the current qualitative evidence base, pointing to a gap warranting further investigation.

## 5 CONCLUSION

This meta‐synthesis highlights the profound psychological, social, and embodied challenges experienced by women with spontaneous POI. Beyond its reproductive implications, POI affects women's identity, relationships, and engagement with healthcare systems. It is not merely a medical condition but a life‐altering phenomenon demanding holistic, woman‐centered care. By amplifying women's voices, this synthesis calls for a paradigm shift, from framing POI as a reproductive disorder to recognizing it as a transformative experience requiring multidisciplinary support. Future efforts must bridge the gap between clinical management and the lived realities of women navigating POI.

These findings should be interpreted as context dependent and reflective of the perspectives represented in the included qualitative studies. Nevertheless, they contribute to a deeper understanding of patient priorities and might help inform the development of patient‐centered outcome measures and supportive care strategies. Further research in diverse populations and healthcare settings is needed to expand understanding of how sociocultural context shapes the lived experience of spontaneous POI.

### 5.1 Recommendations for clinical practice

Table 4 translates the five themes that emerged from this study into practical implications for clinicians managing women with spontaneous POI.

**TABLE 4:** Summary of key clinical implications for clinicians managing spontaneous POI.

Theme	Key finding from women's perspectives	Clinical implication
1. Emotional and psychological impact	Women experience grief, depression, self‐identity disruption, and shame, often exacerbated by societal stigma.	Routinely screen for emotional distress at diagnosis and follow‐up. Offer referral to psychological support or peer support groups. Normalize grief as a valid response, not a pathological one.
2. Physical and embodied experiences	Women report premature aging, fatigue, affected sexuality, and fertility loss as core embodied concerns.	Proactively discuss sexual health (dyspareunia, libido, vaginal dryness) and fatigue management. Avoid assuming these are secondary or less important.
3. Social and relational challenges	POI strains intimate relationships, creates workplace adversity, and leads to isolation due to life‐stage mismatch.	Inquire about relationship support needs and workplace adjustments. Consider providing a letter for employers recommending reasonable accommodations (e.g., flexible hours).
4. Diagnostic and treatment challenges	Diagnostic delays, clinician dismissal, and ambivalence toward HRT and fertility treatment are common.	Use sensitive, non‐dismissive communication (avoid “you're too young”). Acknowledge diagnostic uncertainty while providing clear next steps. Discuss HRT benefits and risks without coercion, respecting cultural and personal values.
5. Coping and adaptation	Women demonstrate resilience through positive reframing, acceptance, and relief at receiving a definitive diagnosis.	Provide a clear diagnosis and written information as early as possible. Facilitate access to peer support networks. Explore and support culturally congruent coping strategies (e.g., family‐ or faith‐ centered, or individual resilience).

### 5.2 Recommendations for future research

- Future studies should explore the experiences of diverse populations, particularly in developing country contexts, to better understand the cultural impacts on women's lived experiences of POI, including how societal norms, stigma, and healthcare access shape their coping mechanisms. Research should also focus on the role of socioeconomic and environmental factors, such as financial barriers or rural–urban disparities, in influencing POI outcomes. Moreover, future studies should explore whether resilience and post‐diagnostic growth differ between women with hereditary/genetic POI compared to those with idiopathic disease, as prior awareness of risk might influence psychological preparation and adaptive strategies. Additionally, studies are needed to evaluate the effectiveness of tailored psychological interventions for POI, examine partner perspectives, and assess the role of peer support networks in fostering coping and adaptation, ultimately informing more holistic and culturally sensitive care approaches.

## AUTHOR CONTRIBUTIONS

Maria Patricia Roman: Conception, planning, data analysis, manuscript writing. Shaurya Aggarwal: Data analysis, reviewing the draft manuscript. Stergios K. Doumouchtsis: Conception, reviewing the draft manuscript.

## FUNDING INFORMATION

No financial support was provided for the conduct of this study.

## CONFLICT OF INTEREST STATEMENT

The authors have no conflicts of interest.

## Supporting information


Material S1

Appendix S1

Appendix S2

Appendix S3

Appendix S4

Appendix S5

## Data Availability

Data sharing is not applicable to this article as no new data were created or analyzed in this study.
