# Supplementary material for: Young bodies, aging ovaries: A systematic review and meta‐synthesis of women's perspectives on spontaneous premature ovarian insufficiency
Source: Int J Gynaecol Obstet. 2026 Jun 12;175(2):619–30. doi: 10.1002/ijgo.71097 (PMC13674854; doi:10.1002/ijgo.71097)
Supplement: Supplementary file 2 [file IJGO-175-619-s001.docx]

**Appendix 5. Critical Appraisal Skills Program Assessments**

| Article / CASP assessment | Was there a clear statement of the aims of the research? | Is a qualitative methodology appropriate? | Was the research design appropriate to address the aims of the research? | Was the recruitment strategy appropriate to the aims of the research? | Was the data collected in a way that addressed the research issue? | Has the relationship between researcher and participants been adequately considered? | Have ethical issues been taken into consideration? | Was the data analysis sufficiently rigorous? | Is there a clear statement of findings? | How valuable is the research? |
| --- | --- | --- | --- | --- | --- | --- | --- | --- | --- | --- |
| Boughton et al., 2002 | y | y | y | y | y | n | y | y | y | y |
| Boughton et al., 2008 | y | y | y | y | y | n | y | y | y | y |
| Golezar et al., 2020 | y | y | y | y | y | ct | y | y | y | y |
| Grootenhuis et al., 2021 | y | y | y | y | y | y | y | y | y | y |
| Halliday et al., 2009 | y | y | y | y | y | ct | y | y | y | y |
| Johnston-Ataata et al., 2020 | y | y | y | y | y | ct | y | y | y | y |
| Johnston-Ataata et al., 2021 | y | y | y | y | y | ct | y | y | y | y |
| Moukhah et al., 2021 | y | y | n | y | y | n | y | y | y | y |
| Moukhah et al., 2023 | y | y | n | y | y | n | y | y | y | y |
| Orshan et al., 2001 | y | y | y | y | y | y | n | y | y | y |
| Singer et al., 1999 | y | y | y | y | y | ct | y | y | y | y |
| Sun et al., 2022 | y | y | y | y | y | y | y | y | y | y |
| Vincent et al., 2024 | y | y | y | y | y | y | y | y | y | y |

Y=yes, n=no, ct=can’t tell.
