# Supplementary material for: Young bodies, aging ovaries: A systematic review and meta‐synthesis of women's perspectives on spontaneous premature ovarian insufficiency
Source: Int J Gynaecol Obstet. 2026 Jun 12;175(2):619–30. doi: 10.1002/ijgo.71097 (PMC13674854; doi:10.1002/ijgo.71097)
Supplement: Supplementary file 4 [file IJGO-175-619-s003.docx]

**Appendix 3. Data collection form**

| **Data extraction field** | **Information extracted** |
| --- | --- |
| Study title or ID | Title / ID |
| Study ID (surname of first author and year first full report of study was published) | 1^st^ author’s surname, year of publication |
| Date form completed | (dd/mm/yyyy) |
| Context and participants | Population description  Setting (including location / social context)  Inclusion and exclusion criteria |
| Study design and methods used | Methodological approach of the study  Specific data collection and analysis methods utilized  Any theoretical models used to interpret or contextualize the findings  The data extraction approach and methods (interview, focus groups, observations, document analysis etc.) |
| Findings | Key themes / concepts identified in the primary studies.  Verbatim quotations of patients  Author’s interpretation of findings |
| Quality of the study | Critical Appraisal Skills Programme (CASP) for qualitative studies |
