# Supplementary material for: Young bodies, aging ovaries: A systematic review and meta‐synthesis of women's perspectives on spontaneous premature ovarian insufficiency
Source: Int J Gynaecol Obstet. 2026 Jun 12;175(2):619–30. doi: 10.1002/ijgo.71097 (PMC13674854; doi:10.1002/ijgo.71097)
Supplement: Supplementary file 5 [file IJGO-175-619-s004.docx]

**Appendix 1. Search query**

‘’( ( TITLE-ABS-KEY ( early AND menopause ) OR TITLE-ABS-KEY ( premature AND menopause ) OR TITLE-ABS-KEY ( early AND onset AND menopause ) OR TITLE-ABS-KEY ( premature AND ovarian AND insufficiency ) OR TITLE-ABS-KEY ( premature AND ovarian AND failure ) ) AND ( TITLE-ABS-KEY ( qualitative AND study ) OR TITLE-ABS-KEY ( qualitative AND research ) OR TITLE-ABS-KEY ( qualitative AND analysis ) ) )’’
