# Supplementary material for: Young bodies, aging ovaries: A systematic review and meta‐synthesis of women's perspectives on spontaneous premature ovarian insufficiency
Source: Int J Gynaecol Obstet. 2026 Jun 12;175(2):619–30. doi: 10.1002/ijgo.71097 (PMC13674854; doi:10.1002/ijgo.71097)
Supplement: Supplementary file 6 [file IJGO-175-619-s006.docx]

**Appendix 4. Summary of analytical themes, contributing codes and patients’ quotations**

|  | **Contributing codes** | **Quotations from contributing studies** |
| --- | --- | --- |
| **Theme 1:**  **Emotional and psychological impact** | Psychological distress | ‘’If you’ve got emotional problems with this (early menopause), that’s quite difficult to deal with.'' ^10^ / ''I was beginning to feel helpless’’ ^12^ / ''The interruption of my period made me feel bad and had a negative energy’’ ^14^ / ‘’ Well, two women in my family, during these 4 years that I had an early menopause, gave birth to their second children, and I had a feeling of jealousy, and it made me think about my problem and I suffered mental illness.’’ ^14^ |
|  | Grief | ‘’(…) sad thing is that I can't have children anymore. Not that I was planning to, it's just that now I can't. I find that quite a grieving process.’’ ^19^ / ‘’I get days where I'm absolutely consumed with grief’’ ^19^ / ‘’ It’s a bit of a bereavement feeling. I realized I hadn’t dealt with the feeling of loss. I dealt with the practical side of things, but emotionally I was crippling myself.’’ ^10^ / ‘’ I feel like I died inside.’’ ^51^ |
|  | Sadness | ‘’ I am so confused about all this and a lot of time I sit down and cry at night when I am alone since I can’t sleep anymore either.’’ ^51^ / ‘’ I can’t enjoy real happiness … why should I be that unlucky?’’ ^12^ / ‘’When I was told that it was menopause I felt very sad, extremely sad because it was confirmation that a certain period of my life had finished.’’ ^35^ |
|  | Depression | ‘’ I even did not want to go home after work, and I was in a depressive mood.’’ ^35^ / ‘’ I was nothing. I wanted to end my life.’’ ^10^ |
|  | Denial | ‘’ I tell lies to my friend about my menstruation’’ ^14^ / ‘’I didn’t believe it [the diagnosis]. I thought: ‘no, no, it must be something else’’ ^10^ / ‘’I’m too young for that’ (…) I was in a bit of denial’’ ^52^ |
|  | Shame | ‘Menopause I feel embarrassed about.’’ ^10^ |
|  | Sense of being cheated | ‘’You just feel empty, you know, you lost that ability, like when you decide you want a family, you don’t want a family, how many kids you want to have. It’s just, you feel like you’re cheated, kind of.’’ ^27^ |
|  | Anger | ‘’ I can’t help but feel angry at the doctor, all those doctors who kept telling me to wait, wait ...’’ ^27^ / ‘’POI makes me angry quickly. I’ll get mad soon’’ ^48^ |
|  | Anxiety | ‘’ I get very nervous when they tell me that someone is pregnant.’’ ^14^ / ‘’ I am anxious;’’ ^12^ |
|  | Helplessness | ‘’I would suddenly go this disgusting feeling-it sort of starts from inside, you know it’s happening and there’s nothing you can do about it.’’ ^10^ / ‘’ Absolutely gutting to discover that you’ve got the potential in 5-10 years’ time to have osteoporosis: to look at yourself in the mirror and say to yourself, how can that be? Here was something you really couldn’t do anything about.’’ ^10^ |
|  | Confidence collapse | ‘’It [menopause] shakes your foundations and your confidence.’’ ^19^ / ‘’ It [menopause] has really affected my self-esteem.’’ ^10^ |
|  | Inadequacy | ‘’ If you say you’re menopausal, you’re not quite 100%, not 100% healthy, not 100% in your mind.’’ ^10^ / ‘’Now that I am disabled and not a perfect woman anymore’’ ^12^ / ‘’ a bit of a sense of not being quite whole as a woman’’ ^11^ |
|  | Self-identity disruption | ‘’You have to redefine yourself-suddenly you’re somebody completely different.’’ ^10^ / ‘’I didn’t feel like a ‘real woman’. I felt inferior’’ ^10^ / ‘’ I just feel there’s something lacking in me-that I’m not quite the person I should be, really’’ ^10^ / ‘’I am too old for my age.’’ ^12^ |
|  | Stigma and concealment | ‘’ I have to hide it because I don’t like anyone to find out about it.’’ ^12^ / ‘’you need to make [people] believe that you are fine while having it with you.’’ ^12^ / ‘’(…) just that stigma that no-one wants to be old‘’ ^52^ |
| **Theme 2:**  **Physical and embodied experiences** | Perception of ageing | ‘’Although I am 37 years old, I do not feel young… I feel aging and I am old’’ ^48^ / ‘’Having to accept that I am officially old no matter what my physical appearance may be. And the fact that I’m on the downhill side, no ifs, ands or buts about it.’’ ^10^ / ‘’People are what they feel themselves to be but I just feel (pause)-60, unappealing and bored.’’ ^10^ / ‘’ I've noticed that my body is beginning to look older’’ ^19^ |
|  | Physical decline | ‘’I've really noticed the muscle tone change too.’’ ^19^ / ‘’Knowing that whatever happened to me dried them up like a bone.’’ ^27^ / ‘’ I feel like I live in a defective, cruel, failing body’’ ^51^ / ‘’my body’s going to disintegrate, my old age starts’’ ^10^ |
|  | Physical limitations | ‘’ I was crawling on my knees by the time I got home from work. I just had no energy. I was very, very tired all the time’’ ^19^ / ‘’ I feel pain deep in my bones. I can’t take long walks’’ ^12^ / ‘’ I can’t go to sleep anymore when this happens, you know, because of the rapid heartbeat and all the sweating’’ ^12^ |
|  | Physical exhaustion | ‘’ an hours light workout in the gym even once a week proved exhausting.’’ ^35^ / ‘’ energy absolutely drained out my toes’’ ^35^ / ‘’I was struggling with my work, because my work's very physical, and I was really exhausted (…) from flushing constantly.’’ ^18^ |
|  | Affected sexuality | ‘’My sex drive is gone.’’ ^51^ / ‘’ I would be incapable of being flirtatious now.’’ ^10^ / ‘’ I could never have an orgasm.’’ ^12^ / ‘’On the sexual level, without the estrogen of course there’s the dryness and so I don’t even know whether that affects desire or not, I guess because you’re not supposed to be menopausal until you are older anyway. And maybe you’re not interested [in sex] when you’re older, I don’t know.’’ ^27^ |
|  | Fertility loss | ‘’ you need to find another sort of identity – not being a mother.’’ ^10^ / ‘’I think more about the fact that this [pregnancy] may never have happened to me’’ ^48^ / ‘’ The attitude that you can’t be of any use apart from bearing children is quite an insult.’’ ^10^ / ‘’to have that choice [becoming pregnant] removed is still, briefly, a moment of (. . .) I don’t want to say loss or sadness. Poignancy? There is a moment of reflection.’’ ^11^ / ‘’Sometimes I think I'm pregnant and I touch my abdomen and talk to an imaginary fetus" ^14^ |
| **Theme 3:**  **Social and relational challenges** | Relational struggle with partner / family | ‘’ From the beginning of my marriage, I was stressed until now because I did not have children. My concern is to have children and that our marriage will fall apart’’ ^48^ / ‘’Getting up before 8 o’clock was near impossible and the children learnt very quickly not to annoy their mother in the morning.’’ ^35^ / ‘’ I’m not even looking to get married, because I know I can’t have the family I would want.’’ ^27^ / ‘’I suddenly thought: ‘my body’s going to disintegrate, my old age starts and he’s only 30’-he won’t desire me anymore, won’t love me anymore, maybe he wants more children, maybe-all those things.’’ ^10^ |
|  | Workplace adversity | ‘’You can’t say ‘Look I can’t go to school [as a teacher] today because I haven’t got the energy’.’’ ^35^ / I worked in a particularly busy restaurant at that time, the possibility of taking breaks was simply not there, and financially I was not able to take any sort of time off work. And I did simply work through it.’’ ^18^ / ‘’I had a very responsible in charge of 7 million dollars’ worth of food I had a lot of pressure and stress if I rang in sick. The boss, a male, couldn’t see why I needed time off ... my world just fell to pieces’’ ^35^ / ‘’(…) moods at work fluctuated from being ‘*too euphoric’* to ‘*too depressed’ ’’ ^18^* |
|  | Life stage mismatch | ‘’ I would like stability first before I have children.’’ ^50^ / ‘’They talked about this stage of life when children are leaving home and here I am, my child had just arrived.’’ ^10^ / ‘’ It’ll be easier once I’m at the age where that’s expected in life.’’ ^10^ / ‘’ It’s because it’s not the norm. Most people say: ‘at my age’, ‘I’m at that age’, and I think, well, I’m not, not at all, I’m half the age.’’ ^10^ |
|  | Isolation | ‘’I like to be at home, to be alone’’ ^48^ / ‘’If you think you’re on your own it makes it worse.’’ ^10^ / ‘’Very few people know ... My partner’s very anti the idea-not because he’s embarrassed, because he thinks it’s totally personal. And that does affect how I talk about it.’’ ^10^ / ‘’ I ask myself in private why it should be like this, but I did not reveal anything to anyone’’ ^14^ / ‘’ If you think you’re on your own it makes it worse.’’ ^10^ / ‘’ I became depressed and thought that I was the only one’’ ^48^ / ‘’ I like to be at home, to be alone’’ ^48^ |
|  | Stereotyping menopause | ‘’ It’s like menopause equals conservative, like the older lady, you know, like quiet. Then [later] I thought, no it's not for me [menopausal clothes].’’ ^19^ / ‘’I thought it meant getting fat, going grey. Now I realize it can happen to anybody, any age.’’ ^10^ |
| **Theme 4:**  **Diagnostic and treatment challenges** | Diagnostic uncertainty | ‘’ With every doctor I saw, I received a different diagnosis.’’ ^48^ / ‘’They thought I was a hypochondriac.’’ ^10^ / ‘’I had felt unwell for quite some time but it was nothing you could put your finger on (…)’’ ^35^ / ‘’You’re having all these symptoms and don’t know what’s wrong with you.’’ ^10^ |
|  | Delayed diagnosis/dismissal by clinicians | ‘’One doctor would say menopause, the next would say perimenopause and the next would say you are fine, there is nothing wrong with you.’’ ^51^ / ‘’I’ve been told over and over again, until quite recently, that everything that’s wrong with me must be all in my mind’’ ^10^ / For months I’d been discounted – whether it be the GP, my husband, or my friends, or even my mother! I felt that I was discounted because, you know, they kept saying ‘You’re too young’ ‘’. ^35^ |
|  | Diagnostic shock | ‘’It was almost like a death verdict. I remember being blasted with it really even though I suspected it.’’ ^10^ / ‘’Dealing with it slowly and making small realizations, because it’s too big a thing to deal with all at once’’ ^10^ / ‘’I really didn’t expect such a thing at all. I was planning (…) a pregnancy. But the exact opposite happened. (…) was the biggest shock of my life.’’ ^48^ / ‘’ It came to me like a blow. I felt awful. I was shocked and frustrated.’’ ^12^ |
|  | Lack of knowledge | ‘’My husband thought POI meant we could no longer have sex.’’ ^48^ / ‘’ Early menopause? I have never heard of this.’’ ^50^ / ‘’ I have no idea what premature ovarian failure or early transition means.’’ ^50^ / ‘’ I did not know before, but when I inclined to have a baby, I later realized that POI result to infertility’’ ^48^ |
|  | Ambivalence toward HRT | “After I started taking HRT, I felt ‘new’ again!!’’ ^51^ / ‘’ I take medicine to regulate my menstruation. My period is regular now and I have no hot flushes. I don’t think of how it affects my health’’ ^12^ / ‘’ I have gained lots of weight since I took the medicines.’’ ^12^ / ‘’ I just was so loathe at such a young age to put myself on that drug path that I worried I may never then come off.’’ ^52^ |
|  | Ambivalence toward fertility (AMH) testing | ‘’If the AMH test were just as easy as a pregnancy test and low in costs, then it would be easier to choose it.’’ ^50^ / ‘’I would definitely do the test. Then I would know what I could expect.’’ ^50^ / ‘’I would not do the test. I live in the moment.’’ ^50^ |
|  | Barriers to fertility treatment | ‘’Because the egg is not mine, I am afraid I will not feel like a mother when she was born’’ ^48^ / ‘’I do not care if I conceive with the donated egg, but its religious issue is important to me. It bothers me.’’ ^48^ / ‘’I think my boyfriend thinks differently about fertility preservation (for example IVF). That is because he is Muslim. According to him, it has to be in a natural way: otherwise, it is not allowed.’’ ^50^ / ‘’accepted the donor egg to save my marriage, but there are some things to worry about. What if the child leaves me because I am not his/her genetic mother?’’ ^12^ |
| **Theme 5:**  **Coping and adaptation** | Positive attitude | ‘’I had a whole year of no periods-fantastic.’’ ^48^ / ‘’After maybe a year or so, it probably had a positive impact on the relationship because we were just able to enjoy life or not worry about whether we might run the risk of having another baby.’’ ^11^ / ‘’I may feel frustrated, but I will not let it affect me’’ ^14^ |
|  | Relief from diagnosis | ‘’I was just so relieved. I could come to work and say ‘I’m sorry I’d been under-performing’-they’re as pleased as I am it’s sorted.’’ ^10^ / ‘’it’s a diagnosis and explains everything that ails you’’ ^10^ / ‘’ Relief-thank God I’m not going mad.’’ ^10^ / ‘’It wasn’t me, it was a medical condition, and you can cope with something if you can give it a name ... when you know that it’s in the textbook it’s reassuring, it’s very comforting and it’s something that can be dealt with’’ ^35^ |
|  | Acceptance | ‘’I have it easier than a lot of people, because of the timing [of early menopause] – I had no interest in having children.’’ ^11^ / ‘’I just got on with being a mother to the kids that I had’’ ^11^ / ‘’It’s probably taken me four to five years to make peace with the fact that I’ve had premature menopause’’ ^52^ / ‘’ I just get on with life, and enjoy it or not, whatever it throws at me.’’ ^10^ / ‘’ I don’t feel I’m losing my feminine side. I’m still me.’’ ^10^ |
