# Supplementary material for: Young bodies, aging ovaries: A systematic review and meta‐synthesis of women's perspectives on spontaneous premature ovarian insufficiency
Source: Int J Gynaecol Obstet. 2026 Jun 12;175(2):619–30. doi: 10.1002/ijgo.71097 (PMC13674854; doi:10.1002/ijgo.71097)
Supplement: Supplementary file 7 [file IJGO-175-619-s002.docx]

**Appendix 2. Prisma flow chart**
